# Targeting the Immune Microenvironment in Acute Myeloid Leukemia: A Focus on T Cell Immunity

**DOI:** 10.3389/fonc.2018.00213

**Published:** 2018-06-13

**Authors:** Adam J. Lamble, Evan F. Lind

**Affiliations:** ^1^Pediatric Hematology/Oncology, Seattle Children’s Hospital, Seattle, WA, United States; ^2^Molecular Microbiology and Immunology, Oregon Health & Science University, Portland, OR, United States

**Keywords:** acute myeloid leukemia, T cells, microenvironment, immunotherapy, tumor antigen

## Abstract

Immunotherapies, such as chimeric antigen receptor T cells, bispecific antibodies, and immune checkpoint inhibitors, have emerged as promising modalities in multiple hematologic malignancies. Despite the excitement surrounding immunotherapy, it is currently not possible to predict which patients will respond. Within solid tumors, the status of the immune microenvironment provides valuable insight regarding potential responses to immune therapies. Much less is known about the immune microenvironment within hematologic malignancies but the characteristics of this environment are likely to serve a similar predictive role. Acute myeloid leukemia (AML) is the most common hematologic malignancy in adults, and only 25% of patients are alive 5 years following their diagnosis. There is evidence that manipulation of the immune microenvironment by leukemia cells may play a role in promoting therapy resistance and disease relapse. In addition, it has long been documented that through modulation of the immune system following allogeneic bone marrow transplant, AML can be cured, even in patients with the highest risk disease. These concepts, along with the poor prognosis associated with this disease, have encouraged many groups to start exploring the utility of novel immune therapies in AML. While the implementation of these therapies into clinical trials for AML has been supported by preclinical rationale, many questions still exist surrounding their efficacy, tolerability, and the overall optimal approach. In this review, we discuss what is known about the immune microenvironment within AML with a specific focus on T cells and checkpoints, along with their implications for immune therapies.

## Introduction

Acute myeloid leukemia (AML) is a clinically and molecularly heterogeneous disorder. Despites its poor prognosis, the treatment of AML remains largely unchanged over the past several decades with high dose chemotherapy remaining the mainstay of therapy (1). This has created an impetus to explore novel therapeutic approaches, such as immune-based therapies or immunotherapies.

The promise of immunotherapy in AML can be traced back to the graft-versus-leukemia effect seen following allogeneic hematopoietic stem cell transplantation (HSCT) (2). This has led to an interest in other immunotherapies, such as bispecific antibodies, chimeric antigen receptor T cells, tumor vaccines, and immune checkpoint inhibitors (ICIs). The presence of a functional T cell population is pivotal to the successful application of immunotherapies for the treatment of malignancies. Conversely, a dysfunctional T cell population may represent a novel therapeutic target for ICIs or other treatments that reinvigorate these cells. In this report, we summarize the current data regarding the functional state of T cells in the AML immune microenvironment.

## Tumor Microenvironment (TME)

The TME can be defined as the cellular environment in which the tumor exists. This environment is made up of endothelial, stromal, and immune cells and plays a key role in the development, propagation, and survival of cancer (3). Characteristics of the TME vary greatly between cancers and can be strikingly different between patients with the same type of cancer. As an example, in renal cell carcinoma, the TME is composed of potentially dozens of types of infiltrating T cells and myeloid cells and this complex mix of cells has predictive ability on disease outcome (4). Large differences can also be observed in the TME between individual metastatic masses within the same patient, making each mass a potentially distinct environment in terms of immune recognition (5). This diversity between and within patients is likely partially responsible for the variability in outcomes.

The TME of hematologic malignancies is substantially different than that of solid tumors. For leukemia, the bone marrow serves as the sanctuary for the majority of leukemic stem cells, but secondary lymphoid organs, such as lymph nodes and the spleen, are also considered to be components of the TME. In lymphomas, the TME appears to be on a spectrum between solid and liquid cancers. The immune microenvironment has been well described in several hematologic malignancies, including Hodgkin lymphoma (HL), acute lymphoblastic leukemia, chronic myeloid leukemia (CML), and chronic lymphocytic leukemia (6–12), but less is known about the microenvironment in AML. Compounding our lack of knowledge is the fact that many studies focused on the immune environment in AML lack sufficient patient numbers or molecular characterization to correctly interpret the reach of observations made in AML, a disease of great molecular heterogeneity. With these caveats in mind, we attempt to summarize the immune observations in AML with a focus on human observations wherever possible.

## T Cells

T cells are an abundant and important component of the immune microenvironment. Within the solid tumor literature, they are termed tumor-infiltrating lymphocytes, and the amount within the TME has been shown to be associated with prognosis (13–19). Similarly, in AML, a study of 66 patients showed an association between bone marrow T cells and clinical outcome. Patients with high percentages of total lymphocytes in their marrow (above 10% of total bone marrow cells) and high T cell percentages (>78.5% of total lymphocytes) were reported to have increased overall survival. The association between survival and T cells was independent of FLT3 and cytogenetic status. High T cell populations also correlated with leukemia-free survival [time between complete remission (CR) and relapse]. These associations were not observed for other parameters such as NK cells or peripheral blast counts (20). While this study needs to be repeated and expanded, it hints at a potential influence of T cells on AML, as observed in other tumor types.

Cytotoxic T lymphocytes (CTLs) are CD8^+^ T cells that play an important role in antitumor immune responses. They are capable of inducing target cell apoptosis through the secretion of granzyme B and perforin. In addition, they have the capacity to secrete large amounts of IFN-gamma, which has an immunostimulatory effect.

CD4^+^ T cells can differentiate into a variety of effector cell types depending on the cytokines present within the microenvironment. They can also act as helper cells that mediate tumor cell killing though B cells, NK cells, and CTLs. In addition, CD4^+^ T cells can differentiate into T-regulatory cells (Tregs), which are important in maintaining self-tolerance. This is accomplished *via* immunosuppressive mechanisms that lead to the inhibition of proliferation and cytokine production of other T cells (21). Elevated numbers of Tregs in solid tumors have been associated with worse outcomes and are attributed to assisting the tumor with immune escape (22).

### Numbers, Distribution, and Activation Status of Immune Cells in AML

There is a paucity of studies detailing the frequency and distribution of T cell within patients with AML, with no clear consensus from the limited number of studies available. One of the most comprehensive phenotypic analyses to date was performed by Le Dieu et al. (23). Comparing the peripheral blood and bone marrow from previously untreated patients with AML (*n* = 36) to that of healthy volunteers (*n* = 17), they were able to characterize the genotype and phenotype by immunophenotyping, T cell receptor (TCR) clonality assessment and gene expression profiling. While the T cell percentages in the bone marrow appeared analogous between the two groups (*p* = 0.58), they found a significant increase in the absolute number of total T cells circulating in the patients with AML (830 × 10^6^ cells/L in healthy versus 1,900 × 10^6^ cells/L in AML, *p* < 0.05). In addition, within this increased number of T cells, there was a higher proportion of CD8^+^ cells demonstrated by the CD4:CD8 ratio (2.5 healthy versus 1.69 AML, *p* = 0.05), and the CD8^+^ population was less clonal (more diverse) compared with the CD4 population (23). This increase in CD8^+^ T cells was also observed by another group that showed a higher number of CD8^+^ cells at diagnosis compared with age-matched healthy donors. Interestingly, this increase normalized following the administration of chemotherapy (24). Le Dieu et al. also demonstrated aberrant T cell activation *via* gene expression profiling (23). This correlates with flow cytometric data from another group that demonstrated an increase of activation markers (HLA-DR, CD69, CD71, and CD57) on T cells at diagnosis when compared with healthy controls (25). Numerous studies have documented elevated numbers of Tregs in patients with AML, which is covered more extensively later in this review (26–30).

The above results are in contrast to groups that have found no differences in the numbers of circulating lymphocytes between patients with AML and healthy individuals (31, 32). There are several explanations for these conflicting results. AML is a phenotypically and genotypically heterogeneous disease, and these studies may not have had sufficient patient numbers to address this heterogeneity. In addition, newly diagnosed patients have different past medical histories, which is likely to influence the overall balance of cells in the immune system.

### Function

The concept of T cell dysfunction, and more specifically, T cell exhaustion was first detailed in chronic viral infections and can be defined as the reduced ability of T cells to proliferate and produce cytokines (33–38). Exhausted T cells can be phenotypically identified by increased expression of several inhibitory receptors [CD244, PD-1, CD160, T cell immunoglobulin domain and mucin domain 3 (TIM-3), LAG-3, and others]. This concept has been further expanded as a possible explanation for immune escape by both solid and hematologic malignancies.

Similar to the conflicting phenotypic results discussed earlier, there is currently no consensus regarding the functional status of T cells in AML. Inconsistencies in functional results may be related to different approaches in defining T cell function. In addition, most assays assess bulk T cell function and may not reveal dysfunction related to antigen-specific T cells that are more central to tumor clearance.

There is some evidence suggesting that T cell dysfunction is present at the time of disease diagnosis. One study found that T cell responses, based on proliferation and cytokine production, following both CD3 stimulation and co-stimulation with anti-CD28, appear impaired. However, this defect in T cell responses could be partially overcome following stimulation with PMA and ionomycin, suggesting dysfunction may be related to the strength of the stimulus. Even in this setting of strong stimulation, the ability of CD4^+^ T cells to produce IFNγ was defective. This impairment of CD4^+^ T cells to produce IFNγ was seen in samples obtained at the time of clinical diagnosis but interestingly this impairment was not present at time of relapse (39). The observed decrease in IFNγ production is in agreement with another report that found reduced levels of IFNγ circulating in the serum of patients with untreated AML compared with healthy controls (40). Using gene expression profiling, Le Dieu et al. found that T cells from patients with AML exhibited global differences in transcription compared with healthy controls (23). Some of the differentially expressed genes were involved in actin cytoskeletal formation. They further demonstrated with an *in vitro* assay that the T cells were impaired in their ability to form immune synapses that are critical for optimal T cell activation (23).

It is likely that T cell function changes based on a patient’s treatment phase and disease status. Using a syngeneic murine model, Zhou et al. were able to identify a subset of CD8^+^ T cells based on phenotype that was deficient in cytokine production and increased in frequency during AML progression (41). This supports the contribution of immune suppression in disease progression and may represent a therapeutic target, such as blocking the checkpoint molecules expressed by these cells. Lichtenegger et al. studied patients who were in CR and found that while their CD4^+^ T cells were reduced in number; their proliferative ability was preserved (42). Conversely, another group found that during periods of chemotherapy-induced leukopenia, T cells were both low in count and also functional capacity following stimulation with an anti-CD3 antibody (43). Functional impairment could be circumvented with optimal co-stimulation through CD28 and led to proliferation values that were similar to healthy controls.

In summary, several defects in T cell function, including proliferation and cytokine production, have been associated with AML. Due to the large mutational and phenotypic variability in AML, further studies will continue to identify T cell defects in patients with AML and identify mutational profiles that result in specific immune landscapes.

## Mechanisms of T Cell Dysfunction in AML

### Tregs in AML

As mentioned earlier, numerous studies have documented elevated numbers and function of Tregs in patients with AML (26–30, 44). The majority of these studies investigated Tregs within peripheral blood, while a few compared Treg frequencies in bone marrow as well. Shenghui et al. compared the blood of patients with newly diagnosed AML (*n* = 182) to age-matched healthy volunteers (*n* = 20). They found that the frequency of Treg cells in the peripheral blood from patients with AML was higher compared with that from the healthy volunteers (9.2 versus 5.44%, *p* < 0.001). In addition, within the same patient cohort, they found a higher Treg frequency in the bone marrow compared with their own peripheral blood (11.9 versus 9.19%, *p* < 0.001). This group also observed that bone marrow-resident Tregs were more immunosuppressive than Tregs from the peripheral blood, based on Treg-induced effects on CD4^+^ T cell division (65.3% undivided CD4^+^ cells versus 58.85% undivided CD4^+^ cells, *p* < 0.05) (28). These findings suggest a preferential accumulation of Tregs both in number and function in the bone marrow of patients with AML, supporting the idea of the bone marrow as an immune privileged niche. By contrast, Wang et al. found similar proportions of Tregs in the bone marrow and peripheral blood of patients with AML (30).

While the number of Tregs appears to be increased at diagnosis, their numbers vary during the course of treatment. Lichtenegger et al. showed reduced Tregs at time of remission but these numbers increased during cytotoxic maintenance therapy (42). Ersvaer et al. showed that Treg frequency decreased from diagnosis compared with after treatment but remained elevated relative to healthy controls (26). Kanakry et al. showed that Tregs are elevated during early recovery from induction therapy and that they remained functionally immunosuppressive (27).

Several studies have suggested a prognostic effect related to the presence of Tregs (28, 29). Mechanistically, this would suggest that Tregs are being utilized by the tumor to suppress normal immune cells. This is supported by a murine model of AML which demonstrated that the frequency of Tregs was increased *in vivo* and that these Tregs had a suppressive function on effector T cells *in vitro*. When the Tregs were depleted, the *in vitro* function of the effector T cells improved, along with treatment outcomes. This suggests that the recruitment of Tregs may be a mechanism of disease persistence (45). These results mirror multiple human studies showing that not only are Tregs elevated at the time of diagnosis of AML but that this increase is associated with a worse prognosis (28, 29).

By contrast, a retrospective study investigating the presence of Tregs in AML during induction chemotherapy demonstrated that higher numbers of these cells in the early phases were associated with better CR and overall survival rates (46). Similarly, a recent systematic review revealed a beneficial effect of increased numbers of Tregs after allogeneic HSCT for AML (47). This prognostic benefit is likely related to the success of the transplant, since immunosuppressive effects of Tregs have a role in minimizing graft-versus-host disease (GVHD).

### Potential Effects of Immune Suppressive Myeloid Subsets on T Cells in AML

Myeloid subsets present in tumors, including macrophages, dendritic cells, and specific immature myeloid subsets known as myeloid-derived suppressor cells (MDSC), have been shown to inhibit T cells. Macrophages can be polarized by signals from their environment into two major subsets, called M1 and M2 macrophages. M1 macrophages are activated by pathogen-associated molecules such as LPS and pro-inflammatory cytokines such as IFNγ and are involved in pathogen clearance and acute inflammatory reactions. M2 macrophages are involved in the resolution of injuries and inflammation and are associated with chronic inflammation. M2 macrophages produce soluble factors such as transforming growth factor beta (TGFβ), arginase, IL-10, and vascular endothelial growth factor that can remodel the local matrix, increase vasculature, and furthermore, inhibit T cell function. There are few studies on the impact of macrophage subsets in AML. One confounding factor for measuring macrophage subsets is that many of the markers used to commonly identify macrophages are often expressed by AML blasts, making the definitive distinction of macrophage or tumor cell difficult.

Acute myeloid leukemia blasts have been shown to differentiate monocytes from healthy donors into an M2-like phenotype in transwell coculture assays indicating that polarization can be achieved by soluble factors alone (48). A small increase in the M2 macrophage population (defined as CD14^+^CD163^+^CD206^+^) in the bone marrow of patients with AML (*n* = 8) compared with healthy donors (*n* = 9) has also been reported (49). In the same study, using mouse models of retroviral oncogene-induced AML, greater numbers of macrophages that promote tumor cell line division were found compared with macrophages from control animals (49). While this study showed a potential for M2 macrophages to support tumor growth in mice, it did not measure the effects of macrophages on T cells.

Myeloid-derived suppressor cells are cells of the myeloid lineage associated with chronic inflammation and cancer. These cells can be divided into two broad categories, polymorphonuclear (PMN-MDSC) and monocyte (M-MDSC), which are phenotypically more similar to granulocytes or monocytes, respectively. These cells can suppress T cell responses though various mechanisms, including the secretion of indoleamine 2,3-dioxygnenase (IDO), arginase, TGFβ, and IL-10. MDSCs can also suppress antigen-specific T cell function by nitrosylation of major histocompatibility complex (MHC)–peptide and TCR complexes, which interrupts T cell target recognition (50). MDSC have also been shown to express immune checkpoint ligands such as PD-L1 that can suppress T cell responses *in vitro* (51). Like macrophage subsets, which have been studied in CML and B cell neoplasms, there is little information regarding MDSCs in AML (52, 53). M-MDSC (defined as CD11b^+^CD14^+/−^ HLA-DR^−^CD15^−^) and PMN-MDSC (defined as CD11b^+^CD14^−^HLA-DR^−^CD15^+^) are elevated in the blood of patients with AML (54). Coculture of AML cells with healthy PBMC has been shown to induce the expansion of a population of immature myeloid cells with an MDSC phenotype that can suppress T cell proliferation and cytokine production (54). There is also an association between Tregs and MDSC numbers in myelodysplastic syndrome (MDS) that correlates with a high risk of transformation to AML, indicating a potential role for MDSC in AML progression (55). While there are studies demonstrating a role for MDSCs in suppressing T cell function in AML, there are also studies showing that they may play a lesser role in this disease. As an example, depletion of MDSCs failed to restore T cell function in a cell line transfer-based mouse model of AML (56). It is clear that the potential impact of both macrophage subsets and MDSC on the function of T cells in the AML microenvironment is not fully established and should be a focus of future studies.

### Soluble Factors

Soluble factors, such as enzymes and cytokines, may help tilt the TME from hostile to supportive for tumor cells by suppressing T cell function. *In vitro* studies suggest the T cell dysfunction seen in AML may be the result of blasts manipulating these soluble factors within the microenvironment (48, 57, 58). Orleans-Lindsay et al. obtained supernatants from both AML cell lines and primary patient samples and then cocultured with isolated T cells. These T cells were unable to proliferate in response to mitogenic or alloantigen stimulation but maintained their cytolytic function (48). Interestingly, when the supernatant was removed, there was partial restoration in the T cell response to mitogenic stimulation (48). Mussai et al. elucidated secretion of arginase II as a specific mechanism of AML blasts creating an immunosuppressive microenvironment (58). They first showed that arginase II activity is significantly raised in the plasma of patients with AML compared with healthy controls (9.9 versus 1.1 μmol; *p* = 0.0001). Furthermore, they showed that when T cells were cultured *in vitro* with the plasma of patients with AML, there was reduced T cell proliferation, which could be relieved *via* arginine replacement. In addition to having a directly immunosuppressive effect on T cells, they showed that AML blasts directly polarize monocytes to an M2-like phenotype, further promoting an immunosuppressive microenvironment (58).

Indoleamine 2,3-dioxygnenase is an enzyme that catalyzes the oxidation of tryptophan to *N*-formylkynurenine. This enzyme is highly expressed in macrophages and activated dendritic cells. The breakdown of tryptophan in the local environment inhibits the proliferative capacity and differentiation of CD8^+^ T cells (59). IDO activity has been shown to result in conversion of CD4^+^ T cells into Treg cells as well as boosting the suppressive capacity of Tregs (60, 61). There is some evidence pointing to a potential role for IDO in AML. First, elevated systemic levels of kynurenine are detectable in patients with AML and have been shown to negatively correlate with overall survival in patients with intermediate risk disease (62, 63). IDO has been measured directly in AML blasts both constitutively and after exposure to IFNγ, indicating that the tumor itself may be responsible for the observed systemic kynurenine levels (64, 65). IDO-expressing AML cells have been shown to direct the conversion of T effector cells into Tregs and, moreover, this effect can be blocked by the addition of the IDO inhibitor, 1-methyl-tryptophan (66). Mansour et al. found a direct correlation between blast IDO expression and an increased percentage of Tregs in patients with AML (67). Therefore, it is possible that the increased Treg population and function seen in AML may be a direct result of IDO expression.

### Immune Checkpoints

Naïve T cells are activated following the mediation of two signals. The first signal is through binding of the antigen-dependent TCR to the MHC molecule on antigen-presenting cells (APCs). The second signal is a co-stimulatory signal also provided by the APCs. The prototypical co-stimulatory molecule is CD28 on T cells with its cognate ligands, CD80 and CD86 expressed on APCs.

Inhibitory checkpoints are molecules in the immune system that function to fine-tune or turn off an immune response. These molecules initiate intracellular signaling events that interrupt activation cascades, thereby leading to decreased T cell proliferation and cytokine production. This process is critical for the establishment and maintenance of peripheral tolerance during normal immune responses. Cancer cells can take advantage of this system by expressing the ligands of these checkpoint receptors to turn off the immune system and avoid destruction. Therefore, blocking interactions between checkpoint molecules and ligands might potentially reverse the tumor effect. The most extensively studied checkpoint molecules are members of the CD28 family, specifically, cytotoxic T-lymphocyte antigen-4 (CTLA-4) and programmed cell death protein 1 (PD-1). ICIs have received FDA approval for the treatment of melanoma, lung cancer, kidney cancer, head and neck cancer, bladder cancer, colorectal cancer and HL (68). HL is of particular relevance because it is a hematologic malignancy that had previously been shown to overexpress the ligands for PD-1 (68). This established a biological basis for the use of PD-1 blockade therapy in HL and now serves as a model for other hematologic malignancies. The use of ICIs after allogeneic hematopoietic stem-cell transplant has been explored with promise as ICIs may be expected to increase or reactivate a favorable graft-versus-leukemia response (69). However, this approach has also been taken with great caution, given the possibility of inducing GVHD. While careful consideration is warranted, initial results indicate that administration of CTLA-4 blockade antibody is possible with tolerable side effects in many cases (70). Importantly, clinical responses were observed, including in AML patients. Clinical trials will continue in the post-allogeneic transplant setting and will yield interesting clinical and mechanistic results [for a concise review of this topic, see Ref. (71)].

### Cytotoxic T-Lymphocyte Antigen-4

Cytotoxic T-lymphocyte antigen-4 normally resides in the cytoplasm in resting T cells and is expressed on the surface of CD4^+^ and CD8^+^ T cells following activation. CTLA-4 shares the same ligands with CD28, CD80, and CD86 expressed on APCs, and represents a key mechanism for the immune system to halt unnecessary or inappropriate T cell activation. By simultaneously outcompeting CD28 and initiating an inhibitory signal, CTLA-4 downregulates TCR activation (72–74). Conversely, CTLA-4 is constitutively expressed on Tregs and provides an activation signal for these cells (75).

CD80 and CD86 have been shown to be upregulated on AML blasts (76–79). Direct engagement with CTLA-4 on normal T cells by these ligands may have the ability to suppress effector T cells. In preclinical models, blockade of CTLA-4 leads to enhanced T cell responses against AML (77, 80). In addition, Laurent et al. has shown that CTLA-4 is constitutively expressed on the surface of AML blasts in patients at the time of diagnosis and in patients with disease resistant to chemotherapy. Engaging CTLA-4 with CD80 and CD86 ligands was able to induce killing of leukemic cells (81, 82).

Based on data from treatment of solid tumors, this effect is likely mediated by both enhancement of effector T cell activity and inhibition of Treg function. The most compelling data to date supporting the role of CTLA-4 in the treatment of patients with AML comes from a phase 1 multicenter study exploring the role of ipilimumab, a CTLA-4 specific ICI, in patients with recurrent hematologic cancer after allogeneic HSCT (70). Twelve of the 28 patients treated had AML. Twenty-two patients received the escalated dose of 10 mg/kg of ipilimumab. Of the five patients who experienced a CR, all of them had AML. Interestingly, four of these patients had extramedullary disease (three with leukemia cutis and one with a myeloid sarcoma). In addition, the patients who responded had fewer circulating Tregs in their peripheral blood following initiation of treatment compared with those who did not respond. These data not only suggest that CTLA-4 blockade may induce a dormant graft-versus-leukemia response but also supports the concept that extramedullary AML may be immunologically distinct compared with AML isolated to the bone marrow and peripheral blood.

### Programmed Cell Death Protein 1 (PD-1)

PD-1 is expressed on the surface of activated T cells. Its ligands, PD-L1 and PD-L2, are expressed on a wide variety of normal immune cells including T cells, monocytes, and dendritic cells. Similar to CTLA-4, when PD-1 and PD-L1/PD-L2 interact, an intracellular signaling cascade is initiated that inhibits T-cell activation (83). As a way of limiting inflammatory responses and preventing tissue damage, most cells are capable of upregulating PD-L1/PD-L2 in the setting of both type 1 and type 2 interferons (α, β, and γ) (84–86). In addition, similar to CTLA-4, PD-1 can be expressed on Tregs and enhance their function (87).

Many cases of AML express PD-L1 and/or PD-L2 and these ligands can be further upregulated in the presence of activated T cells, primarily by the production of IFNγ (88–90). Whether these ligands are constitutively expressed by the leukemia cells or it is an adaptive response to immune pressures, expression by AML blasts has been shown to be associated with a poor prognosis (91). Consistent with this, PD-1 expression has been found to be significantly higher in patients with AML at relapse compared with healthy controls (39). Another study investigating expression levels of activation markers on T cells showed an increase in the percentage of PD-1-positive CD8^+^, but not CD4^+^ T cells, in the blood at diagnosis and after attaining a remission when compared with healthy controls (92). These results establish T cell activation in AML but larger studies are required to carefully identify the functional impact of PD-1 expression on T cells in patients with AML.

Murine models have shown the immunosuppressive capabilities of PD-1 within AML. PD-1 knockout mice injected with an AML cell line had slower AML progression and longer survival compared with wild-type mice (56). Blockade of PD-1 in this model was shown to result in lower AML burden and longer survival than control mice. These data implicate PD-1 as a mechanism of immune escape and represent a therapeutic target (93).

In a phase 1b/2 trial, adult patients with relapsed or refractory AML received treatment with the PD-1 ICI, nivolumab, and azacitidine, a hypomethylating agent, in combination. Of the 53 patients treated, 11 (21%) achieved a CR or CR with incomplete hematologic recovery. The rationale for combining anti-PD-1 therapy with a hypomethylating agent originates from data showing upregulation of PD-L1 and PD-L2 mRNA in CD34-positive cells from patients with AML following treatment with a hypomethylating agent and is discussed in further detail below (94).

### T Cell Immunoglobulin Domain and Mucin Domain 3

T cell immunoglobulin domain and mucin domain 3 is a negative regulatory receptor expressed on CD4^+^ and CD8^+^ T cells, Tregs and dendritic cells. There have been several binding ligands that have been identified for TIM-3, including HMGB1, phosphatidylserine, and galectin-9 (Gal-9) (95). Interaction of TIM-3 on Th1 CD4^+^ T cells results in death of the T cell, thus limiting IFNγ-dependent immune reactions (96). Co-expression of TIM-3 and PD-1 on tumor-infiltrating T cells in solid tumor models marks an exhausted T cell population that can be reactivated if both PD-1 and TIM-3 are blocked (97). TIM-3 and its ligand Gal-9 have been identified as a potential target being expressed on AML blasts and leukemic stem cells (98). Focusing on T cells in AML, there is a reported increase in the percent of TIM-3 expressing CD8^+^ T cells circulating in the blood compared with healthy donors (5.90 ± 4.91 versus 0.96 ± 0.54%) (99). Although a small study, there appears to be an association with a high percentage of TIM-3 expressing T cells in patients with AML who relapse after allogeneic HSCT compared with those who remain in extended remission, indicating that there is a role for functional T cells in killing AML cells. TIM-3 and PD-1 double positive T cells isolated from the blood of patients with AML failed to produce cytokines after stimulation with wither mitogens or stimulation thought the TCR (100). In a murine model of AML, T cells co-expressing PD-1 and TIM-3 were found to have reduced production of IFNγ, TNFα, and IL-2. Blocking either of these receptors individually was not sufficient to restore function but combined blockade yielded increased tumor rejection and improved survival (41). These observations have resulted in a clinical trial with three arms; decitabine plus anti-PD-1, decitabine plus anti-TIM-3 and a combined arm with decitabine plus PD-1 and TIM-3 blockade (NCT03066648). The accumulation of data to date indicates that TIM-3 will continue to be a promising target on both AML cells and tumor-associated T cells.

### T Cell Immunoglobulin and Immunoreceptor Tyrosine-Based Inhibitory Motif Domain (TIGIT)

T cell immunoglobulin and immunoreceptor tyrosine-based inhibitory motif domain is a co-inhibitory receptor expressed on activated T cells, Tregs and NK cells. Like TIM-3, CTLA-4, and PD-1, TIGIT has multiple ligands. Engagement of TIGIT occurs by binding CD155, CD112, or CD226 (DNAM-1) (101, 102). TIGIT has been shown to be upregulated on CD8^+^ T cells in AML and is associated with primary refractory disease and relapse post-transplant (103). A small increase in a population of PD-1^+^TIGIT^+^CD8^+^ T cells has been observed in the blood of patients with AML when compared with healthy controls. This population produces slightly less IFNγ and TNFα compared with the same population isolated from healthy donors when stimulated with anti-CD3 and anti-CD28 (104). The ligands CD155 and CD112 appear to be expressed, possibly at elevated levels, in AML blasts (103, 104). Therefore, TIGIT might contribute in mediating functional abnormalities of T cells in the AML microenvironment. As with other ICIs, studies on the potential therapeutic use of TIGIT blockade are promising and will undoubtedly be the focus of future research.

When used alone, ICIs may permit the immune system to perform its normal function of tumor clearance. When used in combination, ICIs have the additional potential of enhancing the effect of other therapies, specifically other immunotherapies.

## T Cell Targets in AML: Antigens and Vaccines

Clinical responses to immune checkpoint blockade or vaccines require tumor antigens that can be recognized by T cells. These antigens can be from various sources, including novel epitopes from non-synonymous coding mutations in genes, developmentally regulated genes with poor tolerance such as Cancer Testis antigens (CT antigens) or virally associated epitopes.

A major class of antigens associated with tumors is derived by DNA mutations. Recognition of these “*de novo*” epitopes requires several events to occur. First, the mutation must code an amino acid change (non-synonymous mutations). This mutation must then be expressed at the RNA and protein levels, which would not occur for all mutations detected by DNA sequencing. The new peptides then need to bind MHC molecules in order to be presented to T cells. Finally, the gene with the mutation must be expressed at sufficient levels by the tumor cells. It is currently believed that antitumor responses from the use of ICIs are related directly to the number of non-synonymous coding mutations present in the tumor. High mutational burden, including those induced by microsatellite instability, has been shown to correlate with response to immune checkpoint blockade (105–107). This is not absolute, however, as not all highly mutated tumors respond and some patients with low mutational burden are able to mount a response. This indicates that, while mutational burden is a major factor involved in response to ICI, there are other factors involved. Recent studies by Schreiber and others have revealed that even when there are hundreds or thousands of DNA mutations in a tumor, only a small number may meet all the abovementioned criteria (108–110). Numerous recurrent mutations and drivers associated with AML have been identified (111, 112). Overall, AML is thought to be of low mutational burden, falling in the lowest quarter of cancer types and therefore may be predicted to respond poorly to ICI therapy (113). There are, however, specific AML subsets that might yield high epitope expression. These subsets include a p53 loss of function with higher mutational events on average, or a complex karyotype with multiple translocations. Another important issue regarding mutations as targets in AML is the diverse clonality of the disease (114). This diversity at the mutational level in a single patient has been associated with resistance to chemotherapy and may present a similar issue for immunotherapies because one clone expressing an immunogenic epitope may not represent the entire tumor.

Expression of developmental antigens has long been recognized in AML. These proteins are not expressed or are expressed at low levels in normal adult tissues. Expression of antigens such as RAGE, MAGE, WT1, and NY-ESO-1 has been identified and studied in AML, and their expression has been associated with improved clinical outcome (115). These proteins are often regulated at the level of promoter methylation and are often expressed in cancers due to defects in epigenetic regulation (116). DNA methyltransferase inhibitors, such as azacitidine and decitabine, may increase expression of these antigens and therefore can potentially be used as a method to increase the antigenicity of AML. In AML cell lines, treatment with either of these agents resulted in increased RNA and protein expression of NY-ESO-1, WT1, and MAGE A1, A2, and A3 (117, 118).

The goal of vaccine strategies is to boost the number and activity of tumor-reactive T cells. Vaccines based on tumor-associated antigens have shown immunological responses to the tumor and in the remission setting may result in more prolonged remissions (119). One target for AML vaccines is WT1. WT1 is a zinc finger containing transcription factor important in the development of the kidney and other organs and is named after its association with Wilms tumor (120). WT1 is highly expressed by AML blasts in approximately 90% of patients and WT1-specific T cells have been shown to be present (121–123). A number of vaccine trials using WT1 peptide vaccines [(124) and summarized in Ref. (125)] demonstrated both safety and clinical responses, but larger studies are necessary to identify the scope of these responses. It is important to consider that even if T cells successfully expand through vaccination, they will still face the same suppressive mechanisms as naturally primed T cells. For this reason, strategies combining vaccination with other therapies such as immune checkpoint blockade are more likely to be effective.

## Increasing T Cell Responses to AML by Targeting DNA Methylation

Hypomethylating agents are a form of epigenetic therapy and have been used to treat hematologic malignancies such as MDS, chronic myelomonocytic leukemia (CMML) and AML for years. The mechanism of action on tumor cells has been assumed to be due to de-methylation and thus increased expression of tumor suppressor genes (126). While these drugs were first appreciated for their ability to suppress leukemia cell counts, recently their effects on the immune system have gained more attention [reviewed in Ref. (127)]. As described earlier, there has been a substantial body of research showing that hypomethylating agents can increase expression of developmental antigens. There is also evidence indicating direct consequences of hypomethylating agents on T and NK cells. The indiscriminate nature of their effects on gene promoters results in a complex series of positive and negative effects on T cells. FoxP3 is the lineage-defining transcription factor for Tregs and is regulated at the level of the promoter *via* methylation. Treatment of T cells with azacitidine results in stable expression of FoxP3 (128–131). Furthermore, azacitidine treatment has been shown to suppress T cell proliferation and cytokine production, resulting in suppression of GVHD in mice (132). A similar result was shown in patients following HSCT where treatment with azacitidine was associated with an increase in Tregs (133). While this increase in Treg frequency was observed in patients with MDS being treated with azacitidine, the Tregs isolated from these patients had reduced suppressive capacity (134). Treatment of patients with AML or MDS with azacitidine also led to elevated PD-1 expression on T cells by demethylating the PD-1 promoter (135). Likewise, azacitidine treatment increased the expression of a series of other checkpoint molecules, including PD-L1 and PD-L2 in cells from patients with MDS or AML (94).

Additional mechanisms for hypomethylating agents to promote generalized inflammation include reactivation of endogenous retroviruses that are then recognized resulting in an IFN response. This response can then synergize with CTLA-4 blockade to induce antitumor immunity (136, 137). Similarly, expression of costimulatory molecules such as CD80 has been shown to be increased by hypomethylating agents as well, resulting in enhanced antitumor immunity in a mouse model of lymphoma (138). Azacitidine treatment results in expanded antitumor T cell recognition in patients with AML and increased TCR repertoire in patients with MDS (139, 140). As a result of this information several clinical trials have been initiated to investigate the potential of hypomethylating drugs and immune ICIs for AML (Table 1).

**Table 1:** Ongoing clinical trials using checkpoint inhibitors in myelodysplastic syndrome (MDS) and acute myeloid leukemia (AML).

Clinical trial identifier	Checkpoint target	Disease subsets	Combined therapy	Phase, status
NCT03381118	PD-1	Elderly AML	Cytarabine, haploidentical donor PBSC	2, recruiting
NCT01096602	PD-1	AML	DC vaccine	2, active not recruiting
NCT01822509	CTLA-4 or PD-1	Heme malignancy including AML post-allo transplant	None	1, recruiting
NCT01919619	CTLA-4	Post-auto SCT leukemia	Lenalidomide	1, recruiting
NCT01953692	PD-1	MDS	None in MDS arm	1, active not recruiting
NCT02117219	PD-L1 PD-L1 + CTLA-4	MDS	Azacitidine	1, recruiting
NCT02275533	PD-1	AML (remission)	None	2, recruiting
NCT02397720	PD-1 PD-1 + CTLA-4	AML	Azacitidine	2, recruiting
NCT02464657	PD-1	MDS + AML	Idarubicin + cytarabine	1/2, recruiting
NCT02530463	PD-1 CTLA-4 PD-1 + CTLA-4	MDS + AML	Azacitidine	2, recruiting
NCT02532231	PD-1	AML	None	2, recruiting
NCT02599649	PD-1	MDS	KIR2DL1/2L3 azacitidine	2, active not recruiting
NCT02708641	PD-1	AML elderly	None	2, recruiting
NCT02768792	PD-1	R/R AML	Cytarabine	2, recruiting
NCT02771197	PD-1	High-risk AML not eligible for hematopoietic stem cell transplantation (HSCT)	Fludarabine Melphalan HSCT	2, recruiting
NCT02775903	PD-L1	MDS + AML	Azacitidine	2, active not recruiting
NCT02845297	PD-1	AML + R/R AML	Azacitidine	2, recruiting
NCT02846376	CTLA-4 ± PD-1	MDS + AML	None	1, recruiting
NCT02890329	CTLA-4	MDS + AML	Decitabine	1, recruiting
NCT02935361	PD-L1	MDS, recurrent AML	Guadecitabine	1/2, recruiting
NCT02936752	PD-1	MDS	Entinostat	1, recruiting
NCT02953561	PD-L1	AML	Azacitidine	1/2, recruiting
NCT02981914	PD-1	AML, MDS	None	1, recruiting
NCT02985554	PD-1	Post-allo SCT leukemia	None	1, recruiting
NCT02996474	PD-1	R/R AML	Decitabine	1, recruiting
NCT03059485	PD-L1	AML, remission	DC/AML fusion vaccine	2, recruiting
NCT03066648	PD-1 TIM-3 PD-1 + TIM-3	MDS + AML	Decitabine	1, recruiting
NCT03092674	PD-1	AML or high risk MDS	Azacitidine, cytarabine, decitabine, and midostaurin	2/3, recruiting
NCT03094637	PD-1	MDS	Azacitidine	2, recruiting
NCT03146468	PD-1	Post-allo SCT leukemia	None	2, recruiting
NCT03154827	PD-L1	AML	CXCR4	1b/2, recruiting
NCT03259516	PD-1	MDS	Azacitidine Cytarabine Sildenafil Melphalan	1/2, recruiting
NCT03286114	PD-1	MDS, AML, ALL	None	1, recruiting
NCT03291353	PD-1	refractory AML	None	1, recruiting
NCT03358719	PD-1	MDS, AML, chronic myelomonocytic leukemia, and refractory anemia	DEC-205/NY-ESO-1 CDX-1401 decitabine poly OC:C	1, recruiting
NCT03390296	PD-L1 OX40 4-1BB	AML	Azacitidine Gemtuzumab Ozogamicin Glasdegib	2, recruiting
NCT03395873	PD-L1	AML	Decitabine	1, recruiting

## Conclusion

All of the recent advances in tumor immune therapy come from decades of basic immunology research focusing primarily on autoimmunity. In fact, autoimmunity and tumor immunity represent opposite sides of the same coin, one representing an overactive immune system and the other a suppressed immune system. By understanding the immunologic mechanisms that lead to disease, we will be better equipped to treat or prevent these diseases. This is highlighted in solid tumors, where immune therapies such as ICI have led to dramatic cure rates in universally fatal diseases. There is also great interest in using ICIs in AML, as evidenced by the number of clinical trials currently underway (see Table 1) (141). Furthermore, by being able to substitute these therapies for traditional cytotoxic therapies, there is a hope of reducing the acute and chronic toxicities associated with chemotherapy. With the development of drugs targeting specific signaling pathways and mutations in AML along with epigenetic modifiers there is great potential in the near future to develop strategies that combine the high rates of response of targeted agents with the durability of immune therapies. Immunotherapies are just now starting to infiltrate the world of AML and the more we learn about the immune microenvironment, the more successful these therapies will become.

## Author Contributions

All authors listed have made a substantial, direct, and intellectual contribution to the work and approved it for publication.

## Conflict of Interest Statement

EL receives research funding support in the form of sponsored research projects by the following entities: Janssen Pharmaceuticals, Celgene, and Amgen. The remaining author declares that the research was conducted in the absence of any commercial or financial relationships that could be construed as a potential conflict of interest.
